# Facile preparation of poly(*N*-isopropylacrylamide)/graphene oxide nanocomposites for chemo-photothermal therapy

**DOI:** 10.1080/15685551.2022.2111854

**Published:** 2022-08-15

**Authors:** Phornsawat Baipaywad, Naeun Ryu, Soo-Seok Im, Ukjae Lee, Hyung Bin Son, Won Jong Kim, Hansoo Park

**Affiliations:** a Biomedical Engineering Institute, Chiang Mai University, Chiang Mai, Thailand; b Department of Integrative Engineering, Chung-Ang University, Seoul, Republic of Korea; c Department of Chemistry, Pohang University of Science and Technology (POSTECH), Pohang, Republic of Korea

**Keywords:** Poly(*N*-isopropylacrylamide), graphene oxide, nanogel, chemotherapy, photothermal therapy

## Abstract

Carbon-based nanomaterials, such as carbon nanotubes, fullerenes, nanodiamonds, and graphene, have been investigated for various biomedical applications, including biological imaging, photothermal therapy, drug/gene delivery, cancer therapy, biosensors, and electrochemical sensors. Graphene oxide (GO) has unique physicochemical properties and can be used to restore conductivity through oxidation. In this study, we developed poly(*N*-isopropylacrylamide) (PNIPAM)-based nanogel systems containing GO for controlled *in vitro* drug delivery. The photothermal effects of the PNIPAM/GO- and PNIPAMAAM/GO-based nanogel systems were enhanced. The release of DOX from the PNIPAM/GO-based nanogel was achieved using the photothermal effect of near-infrared irradiation. Using a Cell Counting Kit-8 assay, the cytotoxicity of all conditions demonstrated that the PNIPAM composite-based nanogels were biocompatible with no significance.

## 1. Introduction

Cancer is a disease that causes death worldwide for more than 10 million people each year [1]. Consequently, there is increased awareness of the dangers of cancer and attempts to develop new technologies for cancer treatment. Chemotherapy is one of the most often used cancer treatments that can kill cancer cells through medicine [2]. However, chemotherapy has a risk of side effects for patients, for example, nausea, hair loss, fatigue, pain, bleeding, etc [3]. Therefore, photothermal therapy has gained use as an alternative treatment because the increased heat that is generated from near-infrared (NIR) radiation leads to cancer cell death [4].

Recently, graphene-related nanomaterials are gaining increasing interest for use in photothermal therapy, whether graphene oxides (GO) or reduced graphene oxide (RGO). GO is a unique, abundant, and low-cost two-dimensional nanocarbon recently produced from graphite [5]. Naturally, GO has a high dispersibility in water and polar organic solvents because the basal plane and edges of graphene consist of oxygen-containing groups [6]. Furthermore, GO has advantageous properties that make it ideal for biological applications, such as easy synthesis, large surface area, good colloidal stability, easily tunable surface functionalization, and good biocompatibility [7,8]. In several studies, GO has been identified as a drug nanocarrier that can improve drug release profiles for cancer targeting, promote cellular uptake and accumulation of chemotherapy in cancer cells, and reduce chemotherapy side effects on normal cells [9]. Here, GO was incorporated into a nanogel to improve the thermosensitivity and colloidal stability [5,6,10]. Various techniques for modifying GO surfaces with polymer chains have been established in recent years, including free radical polymerization, atom transfer radical polymerization, reversible addition fragmentation-chain transfer, and coupling processes [6,11]. *N*-isopropylacrylamide (NIPAM) was used as a monomer in the creation of poly(*N*-isopropylacrylamide) (PNIPAM)/GO-based nanogels because it allows for easy modification of the functionalization of their surface. To improve the lower critical solution temperature (LCST), PNIPAM-based nanogels can be incorporated with various comonomers, for example, allylamine (AAM) [12–16], acrylic acid [17], etc. In general, PNIPAM can respond to temperature changes in the environment across its LCST of approximately 32°C [5,6,10], while modified PNIPAM can change the LCST towards body temperature [17]. The PNIPAM-based nanogel is hydrophilic below the LCST and becomes hydrophobic when it is above the LCST [5,10,18], which might enhance hydrophobic drug loading stability and increase release efficiency. In addition, it is a challenge to study photothermal effects on copolymerization-based nanogel composites that have never been reported before.

In this study, we report an enhanced photothermal effect of NIR irradiation on PNIPAM/GO and poly(*N*-isopropylacrylamide)-allylamine (PNIPAMAAM)/GO nanocomposites *in vitro*. The chemo-photothermal concept was also used to investigate the release behavior of doxorubicin (DOX) from PNIPAM/GO-based nanogels. Furthermore, the *in vitro* cytotoxicity of the PNIPAM/GO hybrid was investigated using a cell counting kit-8 (CCK-8) assay to determine the relative cell viability. PNIPAM/GO-based nanogels with photothermally controlled drug delivery systems have the potential for cancer treatment.

## 2. Materials and methods

### 2.1 Materials

The monomer NIPAM was recrystallized from hexane and dried using a vacuum before use. The crosslinker *N,N’*-Methylenebisacrylamide (BIS), allylamine (AAM), ammonium persulfate (APS), and sodium dodecyl sulfate (SDS) were purchased from Sigma-Aldrich and used as received. The nano GO powder was purchased from Graphene Supermarket (diameter around 90 to 200 nm). Water used in all reactions was purified to a resistance of 18 MΩ and filtered through a 0.22 µm membrane to remove any impurities.

### 2.2 Preparation of PNIPAM, PNIPAMAAM, PNIPAM/GO, and PNIPAMAAM/GO-based nanogels

**Scheme 1.**
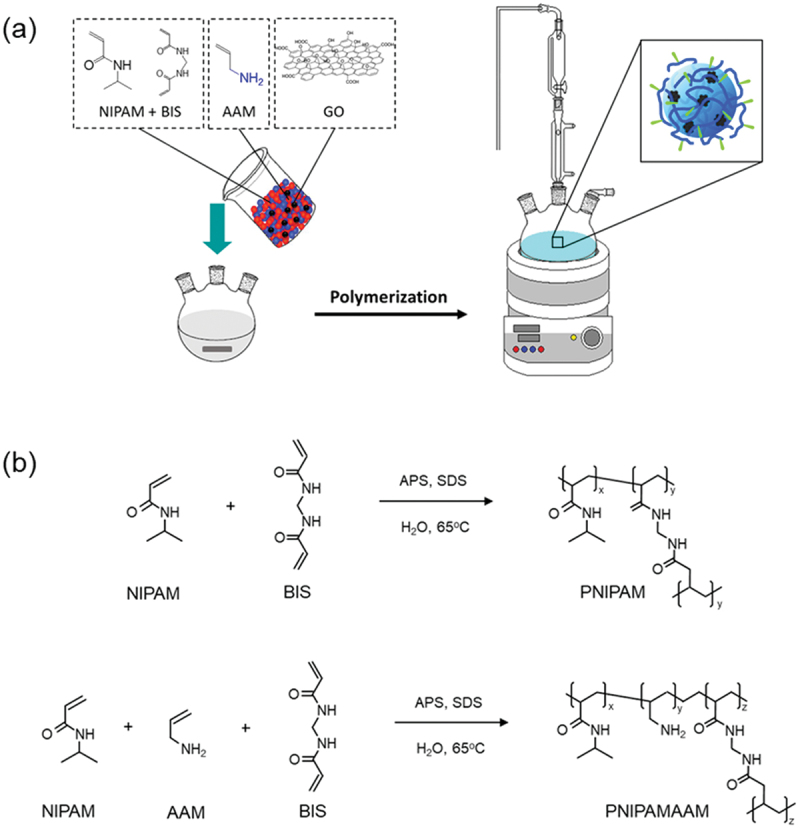
Molecular structures of NIPAM, BIS, AAM, and GO, (a) the synthesis procedure of nanogel homopolymer and hybrid nanogel using conventional radical polymerization methods and (b) the formation of nanogel.

NIPAM: *N*-isopropylacrylamide; BIS: *N,N’*-Methylenebisacrylamide; AAM: allylamine; SDS: sodium dodecyl sulfate; GO: graphene oxide.

The nanogel homopolymer (PNIPAM and PNIPAMAAM) and hybrid nanogel (PNIPAM/GO and PNIPAMAAM/GO) particles were prepared by the conventional radical polymerization method, as shown in Scheme 1(a) [19]. The formation of PNIPAM and PNIPAMAAM-based nanogel is shown in Scheme 1(b). These polymer nanoparticles were used as the templates for encapsulating GO nanopowders within the nanogel particle matrixes. Specifically, a solution containing the monomer (NIPAM), comonomer (AAM), crosslinker (BIS), GO (concentration: 2.5 × 10^−4^% w/v), and surfactant (SDS) was mixed in 195 mL purified Milli-Q water and placed in a three-neck round-bottom flask with an inlet for argon. The solution was bubbled with argon for 1 h and heated to 65°C. Then, 5 mL of APS (0.024 g/mL) was added to the reaction mixture to initiate the polymerization process for 4 h. Argon gas was used throughout the reaction to remove any oxygen, which can intercept radicals and disrupt the polymerization. At the end of the 4 h period, the solution was purified by dialysis against deionized water for 7 days. Finally, nanogels were also carried out of water by the freeze-drying method (lyophilization and vacuum drying) for 48 h to improve the long-term stability of colloidal nanoparticles and were stored at room temperature for later use.

### 2.3 Characterizations

The size and thermoresponsive behavior of the nanogel and hybrid nanogel were investigated by dynamic light scattering using a laser particle analyzer system (NANO ZS, Malvern Instruments Ltd., UK). The light source of the He-Ne laser was used at 633 nm and the measurement angle was 173°. ζ-potential measurements were carried out using highly diluted colloidal dispersions at 25°C. Raman spectra of GO, PNIPAM, PNIPAM/GO, and PNIPAMAAM/GO hybrid-based nanogels with different concentrations of GO were recorded under the following specific conditions: 640 nm laser excitation wavelength, 1 mW power, 1 µm beam diameter, 1 s collection time, and 10 times averaging micro mode. The morphologies of the PNIPAM- and PNIPAM/GO-based nanogels were investigated using energy-filtered transmission electron microscopy (Carl Zeiss: LIBRA®120, Germany). The diluted aqueous dispersions of the nanogels and nanogel hybrids were dried on a carbon-coated copper grid. Finally, 2% uranyl acetate solution was dropped onto the sample before examination. For photothermal effect analysis, we followed the protocol described by Kim et al. [20]. In brief, the PNIPAM/GO hybrid-based nanogel was diluted in deionized water (1 mg/mL) and irradiated using an 808 nm laser (diode laser, JENOPTIK unique-mode GmbH, Germany) on 0.9 cm diameter spots at 4 W/cm^2^. During NIR irradiation, the temperature of the solution was measured every 20s for 6 min using a thermocouple connected to a digital thermometer (Lutron Thermometer TM-917, Taiwan).

### 2.4 Drug loading and photothermally triggered drug release

DOX was loaded onto the PNIPAM/GO-based nanogel (0.01 mg/mL) and stirred at room temperature for 12 h. Dialysis was performed to remove unbound DOX molecules. The PNIPAM/GO-DOX-based nanogel solution was incubated under NIR irradiation (808 nm laser at 4 W/cm^2^) for predetermined times (0–3 h). The release of DOX was estimated by measuring the fluorescence at 550 nm.

### 2.5 CCK-8 cell viability test

The viability of human adipose-derived stem cells (hASCs, obtained from the CHA Medical Center) and breast cancer cells (MDA-MB-231) was measured using a Cell Counting Kit-8 (CCK-8, Dojindo). Cultured hASCs and MDA-MB-231 cells were seeded in 96-well plates at a density of 1 × 10^4^ cells/well in medium and incubated for 24 h (37°C, 5% CO_2_, and 95% humidity). The untreated control, Triton^TM^ X-100 treated control, and PNIPAM/GO nanocomposite-based nanogel were dispersed in Dulbecco’s Modified Eagle Medium (DMEM), added to the cells, and cultured for 24 and 48 h. The results were quantified relative to the negative control by considering 100% cell viability. The cytotoxicity test followed the protocol described in a previous study, and all experiments were performed in triplicate [21]. The cultured media in the 96-well plates were removed from the samples and washed with Dulbecco’s phosphate-buffered saline (DPBS). The cells were then treated for 4 h with a 5% v/v CCK-8 solution in DMEM. Finally, absorbance was measured at 450 nm using a microplate reader (Synergy^TM^ H1, BioTek Instruments Inc.).

### 2.6 Cell imaging experiments

hASCs and MDA-MB-231 cells were seeded in cell culture slides in four-well plates at 2 × 10^5^ cells/well and cultured for 24 h. After the attachment period, hASCs cells were incubated with PNIPAM/GO-rhodamine B (RhB)-based nanogels, while MDA-MB-231 were incubated with PNIPAM/GO-DOX-based nanogels for 24 h in the medium. After removing the medium, the cells were fixed with 4% paraformaldehyde for 1 h. We then added 1% Triton^TM^ X-100 1 h and washed the wells three times with DPBS to remove the unbound compounds. The cell nuclei were stained with 4′,6-diamidino-2-phenylindole (DAPI) for 30 s and washed three times with DPBS. The cells were observed using confocal laser scanning microscopy (LSM880, Carl Zeiss Microscopy, USA).

### 2.7 Statistical analysis

Data are presented as the mean ± standard deviation. The variance and multiple-comparison data between the control and sample groups were compared by two-way analysis of variance. Statistical significance was set at *p* < 0.05.

## 3. Results and discussion

The dried nanogel and nanogel containing GO were redispersed in water (Figure S1) before being used. PNIPAM showed good dispersion with milky white, while PNIPAM/GO changed the color to brown, which indicated GO was embedded in nanogel. Dynamic light scattering was used to assess the swelling behavior of nanogels containing GO. The hydrodynamic size of the PNIPAM hybrid-based nanogel was determined at temperatures between 25°C and 37°C, as shown in Table 1. At 25°C, the hydrodynamic diameters of the PNIPAM- and PNIPAM/GO-based nanogels were 471 nm and 297 nm, respectively. The resulting size decrease could be due to PNIPAM’s strong binding affinity with GO via the carboxylic groups, which causes PNIPAM to contract [22]. The nanogels had grown in size and volume by swelling in the solution through their hydrophilic properties; however, when the temperature was raised to 37°C (above the LCST of ~32°C), they transitioned to a hydrophobic state and shrank in size and volume [23,24]. The temperature-responsive behavior of PNIPAM was maintained regardless of the presence of GO in the hydrogel network. The nanogel in water was confirmed to have a narrow size distribution and excellent dispersity [21] with PDI values less than 0.6. ζ-potential measurements of the PNIPAM-based nanogel revealed a small negative charge, which was attributed to the addition of an initiator to the system [25,26]. The negative charge increased to −10.3 mV when GO was added to PNIPAM due to the carboxyl group (-COOH) and hydroxyl group (-OH) on the surface of GO.

**Table 1.** Dynamic light scattering measurement and ζ-potential results of the PNIPAM-based nanogels.

Sample	25°C			37°C		ζ-potential (mV)
	Size(nm)	PDI		Size(nm)	PDI	
PNIPAM	471	0.30	173		0.05	−0.83
PNIPAM/GO	297	0.58	144		0.25	−10.3

**Figure 1.**
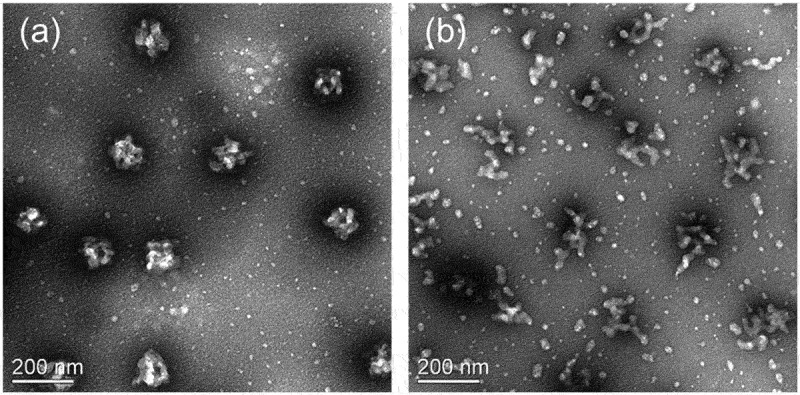
Morphologies of PNIPAM hybrid-based nanogels (a) PNIPAM and (b) PNIPAM/GO.

Transmission electron microscopy (TEM) was used to examine the morphology of the PNIPAM-based nanogels. As shown in Figure 1, PNIPAM-based nanogels showed a round shape with a rough surface (diameters around 138 nm), while the diameters of the PNIPAM/GO-based nanogels were reduced after GO was embedded in the nanogels. However, the TEM images did not indicate the true dimensions of the collapsed gel spheres because of the considerable deformation during drying [27].

The confirmation of the chemical bonding of PNIPAM/GO-based nanogels was observed by FT-IR (Figure S2.). The absorption bands at 3287.0 and 2976.6 cm^−1^ are attributed to the stretching vibration of the – NH groups and C–H stretching (originating from the C–H bond of the PNIPAM chain) [28]. The strong peak at 1642.0 cm^−1^ corresponds to the stretching vibrations of the C = O band, whereas the peak at 1540.8 cm^−1^ represents the stretching vibrations of the C = O band and deformation of the N–H band, which can be ascribed to the typical vibration of O = CNH functionalities of the PNIPAM segment [11]. The absorption peaks at 1456.9 and 1377.8 cm^−1^ correspond to the – NH_2_ groups and the bending vibrations of the isopropyl C–H groups, respectively [28]. Furthermore, the absorption band at 1055 cm^−1^ is attributed to C–O [8], which indicates the GO was encapsulated in PNIPAM-based nanogels.

**Figure 2.**
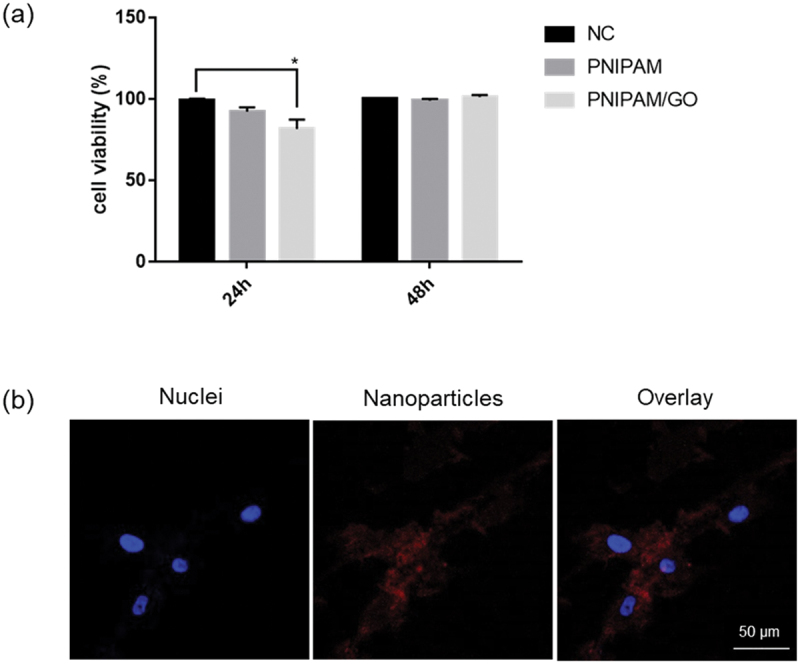
(a) Cytotoxicity of PNIPAM-based nanogels. Human adipose-derived stem cells were treated with 100 µg of PNIPAM and PNIPAM/GO and the cytotoxicity was determined using a CCK-8 assay. (b) Confocal microscopic images of the cells after incubation with PNIPAM/GO-loaded RhB fluorescence nanoparticles at 37 °C for 24 h. Red denotes nanoparticles and blue denotes nuclei. NC: negative control; RhB: rhodamine B.

The toxicity of the PNIPAM-based nanogels was determined using a CCK-8 assay. The results showed that all conditions had low cytotoxicity (<20% cell death) after 24 and 48 h. The only exception was the PNIPAM/GO composite, which showed slight toxicity (>20% cell death) due to GO in the nanogel after 24 h. However, cell viability was recovered after 48 h, as shown in Figure 2a. RhB was used as a fluorescent marker to label particles for cellular uptake. It was introduced into hASC cells via the PNIPAM/GO-based nanogel and cultured for 24 h, while the nucleus was stained with DAPI. Figure 2b shows the cellular uptake and localization of the NIPAM/GO-based nanogel in the cell membrane of hASCs in red. These nanogels end up internalized into cells and selectively distributed in the lysosomes around nuclei.

**Figure 3.**
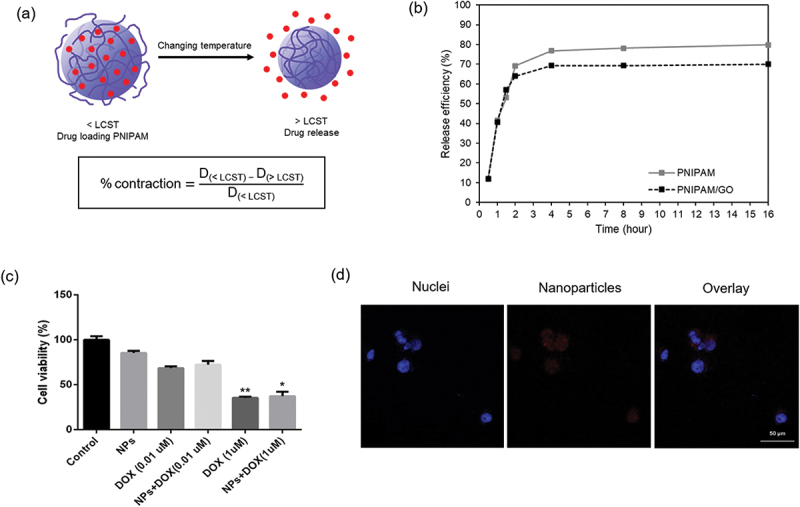
(a) The role of drug release from PNIPAM-based nanogels upon change in temperature, (b) DOX release profile from PNIPAM/GO-based nanogel *in vitro*, (c) Cytotoxicity of PNIPAM/GO-based nanogel. Breast cancer cells (MDA-MB-231) were treated with loaded DOX in PNIPAM/GO-based nanogel (*p* < 0.05), (d) Confocal microscopic images of MDA-MB-231 cells after incubation with PNIPAM/GO-loaded DOX fluorescence nanoparticles at 37 °C for 24 h. Red denotes nanoparticles and blue denotes nuclei. D: diameter. LCST: lower critical solution temperature. NPs: nanoparticles. DOX: doxorubicin.

Temperature sensitivity is the most important factor in drug delivery systems (Figure 3a). This has a direct influence on the swelling behavior of the nanogel for release, as shown in Table 1. The temperature effect on drug release through the PNIPAM/GO-based nanogels was investigated using incubator shaker at 37°C. The results are shown in Figure 3b, where the DOX release profile was encapsulated in the nanogels. PNIPAM and PNIPAM/GO-based nanogels showed DOX release approximately 80% and 70% in 16 h, respectively. After 2 h, DOX-loaded PNIPAM started to show a higher release efficiency over DOX-loaded PNIPAM/GO due to DOX being released out when nanogels became hydrophobic. The release efficiency of PNIPAM was shown to be higher than PNIPAM/GO, which is attributed to the contraction ratio of nanogel. PNIPAM illustrated the shrinkage at 1.72, while PNIPAM/GO was decreased to 1.06. Figure 3c shows that the significant cell viability of the PNIPAM/GO-DOX formulations decreased when DOX was delivered and released, while PNIPAM/GO had no significant toxicity. The application of 1 μM free DOX to MDA-MB-231 cells resulted in approximately 60% cell death after 24 h, because free DOX can pass through the cytoplasmic membrane and diffuse into the nuclei [29]. The delivery of 1 μM DOX-loaded PNIPAM/GO showed a percentage of cell toxicity similar to that of free DOX; approximately 55–60% cell death with 45% viability. These DOX-modified nanogel composites have the potential to be used for chemo- (drug release) and photothermal (heat generation) therapy. They serve as therapeutic agents, opening up new possibilities for cancer therapy in the future. After that, we also observed the cellular uptake of DOX loading nanogels on MDA-MB-231 cells through confocal microscopy. The results showed that MDA-MB-231 cells became round when cells were treated with 1 *µ*M of DOX for 24 h, which indicates the typical characteristics of apoptosis [30]. The red fluorescent signal originated from DOX in the nanogel and the blue signal originated from the nuclear dye, DAPI, as shown in Figure 3d.

**Figure 4.**
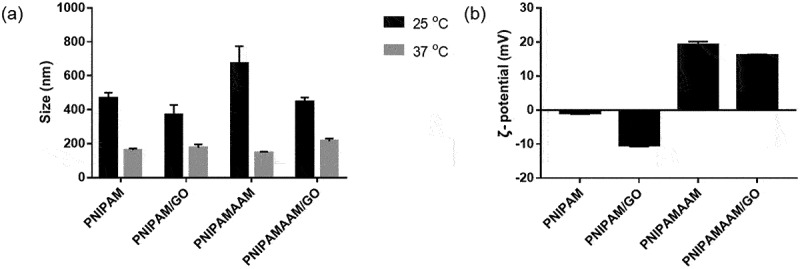
(a) Size averages based on temperature. (b) ζ-potentials of PNIPAM hybrid-based nanogels.

We further examined the properties of PNIPAMAAM-based nanogels encapsulating GO at different temperatures. Figures 4(a,b) show the variation in the size and ζ-potential of the nanogels. The thermoresponsive swelling behavior of the PNIPAMAAM-based nanogels was maintained after the addition of the comonomer to the system. At 25°C, the size of the PNIPAMAAM-based nanogel was larger than that of the PNIPAM-based nanogel; however, when the temperature was increased to 37°C, the shrinkage trend of PNIPAMAAM showed the highest contraction ratio. These results might have come from the loose network of the amine group. Meanwhile, at 25°C, the PNIPAMAAM/GO hybrid nanogels exhibited a small size, and after increasing the temperature to 37°C, the size was larger than that of the pure nanogels, possibly due to GO blocking the shrinkage of the PNIPAMAAM network. The ζ-potential values of the PNIPAMAAM-based nanogels were positively charged at approximately 20 mV due to the addition of comonomers [31], whereas the value of the PNIPAMAAM/GO hybrid nanogels was slightly decreased owing to the functional groups (–COOH and – OH) on the surface of GO. The chemical bonding of PNIPAMAAM/GO-based nanogels was confirmed as shown in Figure S3. All the absorption peaks that appear have already been described in the PNIPAM/GO-based nanogels. The C–O bonding was also observed around 1062.5 cm^−1^, which confirms the GO within the PNIPAMAAM.

**Figure 5.**
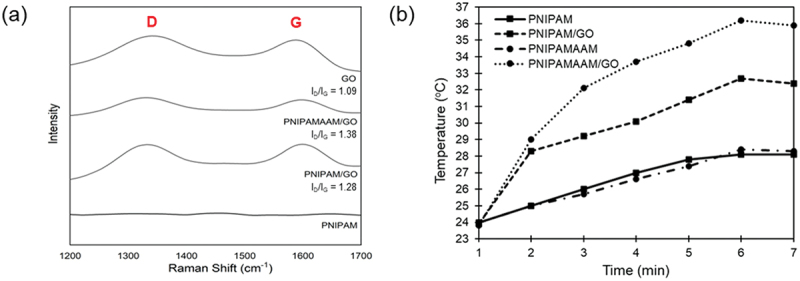
(a) Raman spectra of hybrid PNIPAM-based nanogels, where D and G denote the characteristic D and G bands of graphene, respectively. (b) The photothermal effects of hybrid PNIPAM-based nanogels.

Two main GO peaks at 1342 cm^−1^ (D band) and 1588 cm^−1^ (G band) in the Raman spectrum were created by the Stokes shift caused by laser excitation [8,32] (Figure 5a). The D band is due to the vibration of carbon atoms of disordered graphite, indicating the formation of sp^3^ carbon in GO, whereas the G band arises from the vibration of the sp^2^ carbon lattice of the graphitic domain [33–35]. The G peaks of the PNIPAM/GO- and PNIPAMAAM/GO-based nanogels shift slightly at 1588 cm^−1^, indicating that PNIPAM and GO interact via the free radical process [36]. The I_D_/I_G_ ratio increased from 1.09 in pure GO to 1.28 and 1.38 for PNIPAM/GO and PNIPAMAAM/GO, respectively, which is attributed to the functionalization of the amide group [36,37].

The heat generation by NIR laser irradiation (808 nm, 4 W/cm^2^) was investigated in the control and hybrid PNIPAM-based nanogel solutions, as shown in Figure 5b. When the samples were irradiated with a heated diode laser, the temperatures of the control solutions of the PNIPAM- and PNIPAMAAM-based nanogels increased slightly (~27°C). After NIR irradiation of the solution of PNIPAM/GO- and PNIPAMAAM/GO-based nanogels, we discovered that the temperatures increased due to the transfer of absorbed energy of GO within the network [5]. The highest temperature was raised from 24°C to 36°C in 300 s of NIR irradiation, confirming the facile control of heat generation in the PNIPAM-based nanocomposite by incorporation of GO and comonomer (positive charge) into the system [38,39]. These results indicate a trend that can be suggested as the energy absorbed by photon-activated graphene-based nanomaterials; however, it is necessary to increase the transfer of absorbed energy in the future for chemotherapy.

## 4. Conclusion

In this study, we synthesized and characterized thermally sensitive PNIPAM/GO-based nanogels for controlled drug delivery via NIR irradiation. PNIPAM/GO-DOX successfully delivered an anticancer agent to cancer cells via the photothermal effect of NIR irradiation. The nanocomposites of the PNIPAM/GO- and PNIPAMAAM/GO-based nanogels exhibited an enhanced photothermal effect upon the addition of GO and the comonomer inside the particle. The cytotoxicity under all conditions was determined using a CCK-8 assay, which demonstrated that the PNIPAM composite-based nanogels were biocompatible with less toxicity. Although GO in PNIPAM-based nanogel is not ideal for photothermal therapy compared to RGO, this study can prove that the comonomer can affect the photothermal properties. Thus, the development of DOX-modified nanogel composites can serve as a therapeutic agent, opening up new possibilities for cancer therapy in the future.

## Supplementary Material

Supplemental Material
