# Supplementary material for: Facile preparation of poly(N-isopropylacrylamide)/graphene oxide nanocomposites for chemo-photothermal therapy
Source: Des Monomers Polym. 2022 Aug 15;25(1):245–53. doi: 10.1080/15685551.2022.2111854 (PMC9397426; doi:10.1080/15685551.2022.2111854)
Supplement: Supplemental Material [file TDMP_A_2111854_SM4108.docx]

**Supplementary Materials:**

**Facile preparation of poly(*N*-isopropylacrylamide)/graphene oxide nanocomposites for chemotherapy**

Phornsawat Baipaywad^1,2^, Naeun Ryu^2^, Soo-Seok Im^3^, Ukjae Lee^2^, Hyung Bin Son^2^, Won Jong Kim^3^, and Hansoo Park^2,*^

*^1^Biomedical Engineering Institute, Chiang Mai University, Chiang Mai 50200, Thailand*

*^2^Department of Integrative Engineering, Chung-Ang University, Seoul 06974, Republic of Korea*

*^3^Department of Chemistry, Pohang University of Science and Technology (POSTECH), Pohang 37673, Republic of Korea*

^*^Corresponding authors: heyshoo@cau.ac.kr (H. Park)

**
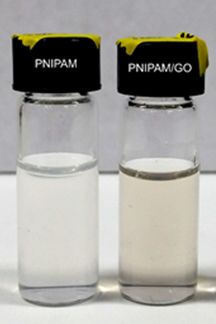
**

**Figure S1.** The photograph of PNIPAM and PNIPAM/GO nanogel solutions.

**
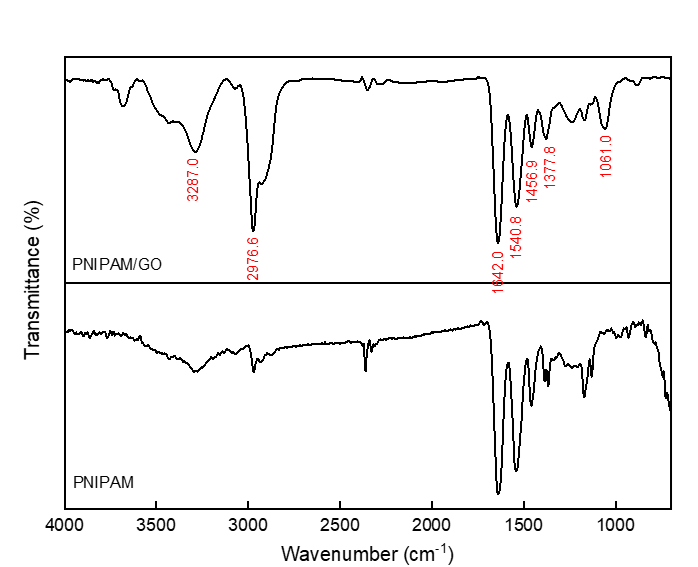
**

**Figure S2.** FT-IR spectrums of PNIPAM- and PNIPAM/GO-based nanogels.

**
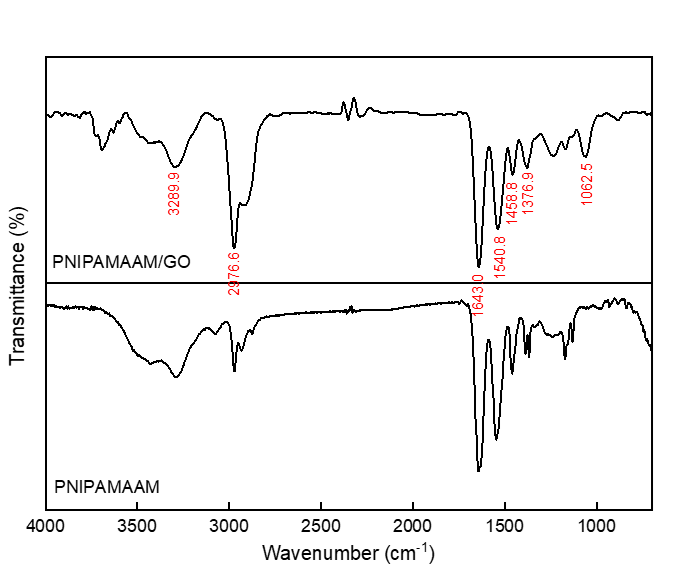
**

**Figure S3.** FT-IR spectrums of PNIPAMAAM- and PNIPAMAAM/GO-based nanogels.
